# Interrelationships of Anxiety, Burnout, Depression, and Insomnia Among Chinese Healthcare Workers Exposed to Workplace Violence: A Network Analysis

**DOI:** 10.1155/da/9815503

**Published:** 2025-12-15

**Authors:** Huixue Xu, Zejun Li, Xin Wang, Min Wu, Tieqiao Liu, Qiuxia Wu

**Affiliations:** ^1^ Department of Psychiatry, National Clinical Research Center for Mental Disorders, National Center for Mental Disorders, The Second Xiangya Hospital of Central South University, Changsha, 410011, Hunan, China, csu.edu.cn

**Keywords:** adverse outcomes, burnout, mental distress, network analysis, workplace violence

## Abstract

Workplace violence (WPV) against healthcare workers (HCWs) is a global public health concern. In China, HCWs face similar challenges; however, the impacts of WPV exposure on their mental health and occupational functioning remain unclear. This study aims to investigate the symptom network of anxiety, burnout, depression, and insomnia (ABDI) and adverse outcomes in Chinese HCWs exposed to WPV. From November 2023 to June 2024, 3361 HCWs with confirmed experiences of WPV were recruited across China through snowball sampling. WPV and ABDI were assessed using the Workplace Violence Scale, Generalized Anxiety Disorder Scale‐7 (GAD‐7), Oldenburg Burnout Inventory (OLBI), Patient Health Questionnaire‐9 (PHQ‐9), and Insomnia Severity Index (ISI), respectively. Data on adverse personal outcomes (low quality of life [QOL] and suicidal ideation [SI]) and occupational outcomes (turnover intention [TI]) were collected. We constructed and visualized networks, calculating strength and bridge strength (BS) indices to identify central and bridge symptoms. In the ABDI network of WPV‐exposed HCWs, exhaustion (EX) emerged as the most central and bridge symptom. Uncontrollable worry (GAD2) and difficulty staying asleep (ISI2) were also central symptoms, while fatigue (PHQ4) and restlessness (GAD5) were identified as secondary bridge symptoms. Disengagement (DEM) strongly predicted TI; EX and sleep dissatisfaction (ISI4) predicted low QOL; and SI was most strongly linked to worthless (PHQ6), motor disturbance (PHQ8), and feeling afraid (GAD7). This study highlights that EX may serve as a priority target for preventing and intervening in psychological distress among WPV‐exposed HCWs, as well as for improving their QOL. Burnout extends beyond workplace‐related issues and is broadly associated with mental health problems and adverse outcomes. Implementing early detection and intervention measures is crucial for preventing and alleviating adverse effects in HCWs exposed to WPV.

## 1. Introduction

Workplace violence (WPV) against healthcare workers (HCWs) is a widely recognized global issue [1–3]. WPV is defined as any act or threat of physical violence, harassment, intimidation, or other threatening behavior that occurs at the work site [4]. HCWs are particularly vulnerable to workplace aggression [5]; estimates indicate they are up to 16 times more likely to experience WPV than professionals in other fields [3]. Furthermore, one in three HCWs has encountered threats of violence, psychological or physical abuse, or sexual harassment from patients or their family members [6]. A systematic review concluded that the global prevalence of WPV among HCWs was 61.9% [7, 8]. WPV exerts extensive negative effects on HCWs, healthcare services, and society at large. It not only elevates the risk of mental distress (including depression, anxiety, burnout, and sleep disturbances [9, 10]) among HCWs but also has a range of adverse personal (e.g., low quality of life (QOL) [11] and suicidality [12, 13]) and professional consequences (e.g., reduced job satisfaction [14], increased turnover intention (TI) [15, 16], and diminished productivity [8]). Additionally, it may lead to higher financial burdens for healthcare organizations and undermine public trust in the healthcare system [17, 18]. Thus, the mental health of WPV‐exposed HCWs demands urgent attention and early intervention.

It is well established that psychological problems are frequently comorbid. Affective events theory (AET) posits that emotions are responses to events within the work environment. Specific events—such as task diversity and interpersonal conflict—trigger positive or negative emotions, while an individual’s emotional stability and current mood moderate the intensity of these emotional responses [19]. For instance, HCWs may experience negative emotions after encountering WPV, and these emotional reactions further influence work attitudes and behaviors, such as TI. Numerous studies using traditional latent variable modeling have consistently demonstrated strong associations between depression, anxiety, burnout, and sleep disturbances among WPV‐exposed HCWs [14, 20–23]. However, latent variable models overlook the specific associations and dynamic interactions among individual symptoms [24]. It is essential to recognize that these conditions comprise multiple sub‐symptoms, which often interact and overlap. A symptom‐based approach, network analysis (NA), may offer new perspectives on the complex interactions among WPV, mental distress, and adverse outcomes. This methodology emphasizes the complexity and dynamism of the research subjects [25]. The interrelationships among variables can be represented as a network of nodes (symptoms) and edges (relationships between symptoms) [24, 26]. Based on the network theory of psychopathology [27], this approach quantifies the significance of each symptom, identifying the “central symptom” that is most closely linked to and influences the other symptoms in the network, as well as the “bridge symptoms” that drive the co‐morbidities of anxiety, burnout, depression, and insomnia (ABDI). These symptoms play a crucial role in triggering and sustaining mental illness networks, making them potential targets for intervention [28, 29]. Therefore, investigating the interrelationship between sub‐symptoms and identifying key symptoms are of great importance [30].

Several studies have explored the network structure of burnout, depression, anxiety, and adverse outcomes among HCWs [31–33]. However, only a limited number of studies have investigated the complex interplay between WPV, mental distress, and related outcomes in healthcare settings. For instance, Yun et al. [34] used mixed graphical models and directed acyclic graphs to examine the primary components and propagating patterns of “mental health–WPV” interactions among training physicians, revealing a strong association between physical violence and psychomotor symptoms. Likewise, Dong et al. [35] identified “depression” and “anxiety” as key symptoms within the network linking WPV, psychopathological symptoms, and deviant workplace behavior. Nevertheless, these studies primarily focused on associations with WPV, often neglecting the mental health consequences following WPV exposure and the specific symptoms driving adverse outcomes. To our knowledge, only one NA, involving 510 nurses exposed to horizontal violence [36], has identified “trouble relaxing” and “startle” as central symptoms within networks of anxiety, depression, and post‐traumatic stress disorder, with “suicidal ideation (SI)” and “restlessness” emerging as key bridge symptoms. To address these gaps, we employed NA to quantify the relationships among mental distress symptoms resulting from WPV in HCWs and to explore their associations with adverse outcomes. The aims of our study were (1) to explore the network structure of ABDI among HCWs exposed to WPV; (2) to identify central and bridge symptoms within this network; and (3) to determine which symptoms are associated with adverse personal outcomes, including low QOL, TI, and SI.

## 2. Materials and Methods

### 2.1. Study Design and Participants

This study was a cross‐sectional survey conducted via an online questionnaire from November 2023 to June 2024. Participants were recruited nationwide using the snowball sampling method. The inclusion criteria were (1) Chinese, aged 18–60 years; (2) currently employed as a physician or nurse in any healthcare institution in China (regardless of department) and with at least 1 year of clinical experience; (3) a score of ≥ 1 on the Workplace Violence Scale (WVS) (indicating exposure to WPV); and (4) ability to understand the questionnaire and voluntary agreement to participate. The exclusion criteria were (1) undergraduate medical students or HCWs working overseas; (2) individuals who had resigned or retired; (3) those working in nonclinical roles (e.g., research); and (4) submission of invalid questionnaires. Participation was anonymous and voluntary. All participants provided written informed consent and could withdraw from the study at any time without consequences. The study was approved by the Ethics Committee of the Second Xiangya Hospital of Central South University (JY20200326).

### 2.2. Data Collection and Quality Control

Data for this study were collected using the Chinese online survey platform Wenjuanxing (https://www.wjx.cn) and the social media platform WeChat. Initially, 40 HCWs from various hospitals across China were selected as “distributors” to share the survey link. They invited their colleagues and peers to participate via WeChat groups and Moments. Participants accessed the survey by clicking the provided link and were encouraged to further distribute it within their own WeChat networks. After clicking the link, respondents were presented with information about the study’s content and purpose and asked to provide consent. Those who selected the option “I have read the informed consent form and voluntarily agree to participate in this survey” were redirected to the main survey page. The survey was designed to require completion of all questions before submission, ensuring no missing data in the final dataset. To validate response quality, several criteria were used to identify invalid submissions: (1) incorrect answers to the “trap” question (“When is Chinese National Day?”) or inconsistent responses to repeated questions; (2) identical answers to all survey questions; and (3) logical inconsistencies (e.g., contradictions between related questions or responses that violated common sense). Additionally, each IP address and WeChat account was limited to one submission to prevent duplicate responses from the same individual.

### 2.3. Sociodemographic and Work‐Related Characteristics

Sociodemographic characteristics assessed in this study included age, sex, marital status, education level, and history of mental health disorders. Work‐related factors examined included professional role (physician or nurse) and professional title.

### 2.4. Workplace Violence

The Workplace Violence Scale (WVS) was used to assess HCWs’ experiences with WPV [37]. The scale comprises five dimensions, each measured by a single item, totaling five items: physical assault, emotional abuse, threats, verbal sexual harassment, and physical sexual harassment. Each item is scored from 0 to 3 points, corresponding to the frequency of violence experienced: 0 points for 0 times, 1 point for 1 time, 2 points for 2–3 times, and 3 points for ≥ 4 times. The total scale score is calculated by summing the scores for each item, with a range of 0–15; higher scores indicate a higher frequency of WPV exposure. In this study, a total score of ≥ 1 was adopted as one of the inclusion criteria, indicating that participants had experienced at least one form of WPV in the past year.

### 2.5. Mental Distress

Mental distress in this study included burnout, depression, anxiety, and insomnia, assessed using the following validated scales.

The Oldenburg Burnout Inventory (OLBI) was used to assess occupational burnout [38, 39]. This scale comprises 16 items evaluating two core dimensions: exhaustion (EX) and disengagement (DEM). Each item is rated on a 4‐point Likert scale, with a total score ranging from 16 to 64; higher scores indicate more severe burnout. The Chinese version of the OLBI has demonstrated good reliability and validity among nurses [40]. In this study, participants were considered to have burnout if their scores on both the EX and DEM dimensions exceeded the respective median scores for each dimension.

Depression and anxiety symptoms were assessed using the Chinese versions of the Patient Health Questionnaire‐9 (PHQ‐9) [41] and the Generalized Anxiety Disorder Scale‐7 (GAD‐7) [42], respectively. Both scales are widely utilized and have shown good reliability and validity among Chinese HCWs [41, 42]. Items are rated on a 4‐point Likert scale (0 = “not at all” to 3 = “almost every day”), with higher scores indicating more severe symptoms. Consistent with previous studies [32], a cut‐off score of ≥ 10 was used to identify clinically relevant depressive and anxiety symptoms.

The Insomnia Severity Index (ISI) [43] was used to assess perceived insomnia severity over the past 2 weeks. The scale consists of seven items, each rated on a 5‐point scale (0–4), with a total score ranging from 0 to 28; higher scores indicate more severe insomnia. The Chinese version of the ISI has been validated and exhibits good psychometric properties [44]. In this study, a total score of ≥ 15 was considered indicative of clinical insomnia [43].

### 2.6. Suicidal Ideation

Current SI was assessed using the ninth item of the PHQ‐9, which asks: *“Thoughts that you would be better off dead, or thoughts of hurting yourself in some way?”* This item is widely used in epidemiological research to evaluate SI [45]. In line with prior studies, we categorized participants as having SI if they scored 1 (a few days) or higher on this item [12].

### 2.7. Turnover Intention

TI was measured with a validated, standard question: respondents indicated the likelihood of leaving their current institution within the next 2 years, using a 5‐point Likert scale (1 = “none” to 5 = “definitely”). This measure has been validated and widely used among physicians [46, 47]. Following previous reports, responses of moderate likelihood or higher (score ≥ 3) were classified as indicating TI [46, 47].

### 2.8. Quality of Life

We used a single‐item linear analog self‐assessment (LASA) question to evaluate overall QOL. This instrument measures overall QOL in the past week on a 0–10 scale, with response anchors ranging from “worst possible” (0) to “best possible” (10). Participants with a score of at least 0.5 standard deviations below the group’s sex‐matched mean were regarded as having low QOL [48]. This measure has been well‐validated and widely used among medical staff [32, 49, 50].

### 2.9. Statistical Analysis

The normality of the variables was assessed using quantile–quantile (Q–Q) plots and the Kolmogorov–Smirnov test. Non‐normally distributed continuous variables were presented as medians with interquartile ranges (IQRs: first quartile, third quartile). Categorical data were expressed as frequencies and percentages. All statistical analyses were performed using R (version 4.3.2). All tests were two‐tailed, with a significance level set at 0.05.

### 2.10. Network Analysis

#### 2.10.1. Network Estimation and Visualization

Since the symptom network included the insomnia symptom cluster, the sleep problems (PHQ3) symptom was removed. The “psych” R package was employed to assess the informativeness of the items, while the “goldbricker” function from the “networktools” package was used to detect redundant items [51]. Items identified as redundant or with low informativeness were considered for removal, but no such items were found in this analysis. We used the “estimateNetwork” function from the “bootnet” package to model and visualize the ABDI network of participants [24]. The Gaussian graphical model was applied with the default “EBICglasso” setting and the “spearman” method [32]. In the network, thicker edges represent stronger correlations, with red edges indicating negative associations and blue edges signifying positive associations [52].

#### 2.10.2. Central and Bridge Symptoms

We utilized the R package “qgraph” to calculate the strength of each node, which quantifies the significance of individual nodes within the network structure. Nodes with higher strength were recognized as central symptoms, potentially playing a key role in initiating and sustaining the network [53]. Additionally, the R package “mgm” was employed to evaluate node predictability in the network [54]. Predictability was defined as the proportion of variance in a node that is explained by all other nodes in the network. This measure reflects the controllability of the network: nodes with high predictability can be influenced by adjusting their neighboring nodes [55]. Furthermore, we computed the bridge strength (BS) index to identify symptoms that connect different domains of the ABDI network [26]. Nodes with higher BS scores were found to have stronger links to symptoms in other areas of distress.

#### 2.10.3. Network Stability and Accuracy

We applied a nonparametric bootstrapping procedure with 1000 bootstrap samples to assess the accuracy of the estimated edge weights in the network. A narrow 95% confidence interval (CI) with minimal overlap suggests that the edge weight estimates are reliable. To evaluate the stability of both strength and BS, we used a case‐dropping procedure. The correlation stability coefficient (CS‐C) measured the stability, with values above 0.5 reflecting a high level of network stability. Furthermore, we conducted bootstrapping on the paired differences of edge weights and the paired differences of centrality values across different nodes to determine if there were significant differences between edges or nodes. The three procedures were performed using the R package “bootnet” [32].

#### 2.10.4. The Association of ABDI With Adverse Outcomes

Finally, we visualized the symptoms significantly associated with SI (PHQ‐9 Item 9) using the “flow” function. Additionally, we separately incorporated the variables “QOL” and “TI” into the ABDI network, visualizing the symptoms directly linked to these two adverse outcomes.

## 3. Results

### 3.1. Sample Characteristics

A total of 3361 HCWs participated in the online questionnaire. Of these participants, 289 answered the trap question incorrectly, 127 provided inconsistent responses to duplicate questions, 35 gave identical answers across all questions, and 15 submitted duplicate entries. After screening, 2895 (86.1%) were deemed eligible for further analysis (Table 1). Participants had a median age of 31 (IQR, 26–38). The majority were female (2033, 70.2%), physicians (2136, 73.8%), married (1602, 55.3%), holding a bachelor’s degree (2090, 72.2%), and had a junior professional title or below (1612, 55.7%). Additionally, 207 (7.2%) HCWs had a history of mental illness. We also assessed the prevalence of WPV and psychopathological symptoms among the participants. Regarding WPV, over 1000 HCWs had experienced two or more types of WPV. Emotional abuse was the most prevalent (84.5%), followed by threats (39.5%), physical assault (32.5%), and verbal sexual harassment (17.0%); physical sexual harassment was the least prevalent (7.7%). For psychopathological symptoms, the prevalence rates of depression, anxiety, burnout, clinical insomnia, low QOL, TI, and SI were 36.1% (*n* = 1044), 21.6% (*n* = 624), 42.8% (*n* = 1239), 19.4% (*n* = 562), 36.9% (*n* = 1069), 22.5% (*n* = 651), and 29.5% (*n* = 853), respectively.

**Table 1:** Sample characteristics of healthcare workers.

Characteristic	Overall, *N* = 2895^a^
Age	31 (26, 38)
Sex	
Female	2033 (70.2%)
Male	862 (29.8%)
Marital status	
Single	1210 (41.8%)
Married	1602 (55.3%)
Divorced or widowed	83 (2.9%)
Education level	
Associate degree or vocational diploma	338 (11.7%)
Bachelor’s degree	2090 (72.2%)
Master’s or doctoral degree	467 (16.1%)
History of mental illness	207 (7.2%)
Professional role	
Physician	2136 (73.8%)
Nurse	759 (26.2%)
Professional title	
Junior level and below	1612 (55.7%)
Intermediate or senior level	1283 (44.3%)
Violence styles	
Physical assault	941 (32.5%)
Emotional abuse	2447 (84.5%)
Threats	1143 (39.5%)
Verbal sexual harassment	491 (17.0%)
Physical sexual harassment	223 (7.7%)
PHQ‐9 scores	8 (5, 12)
Depression symptoms	1044 (36.1%)
GAD‐7 scores	6 (3, 9)
Anxiety symptoms	624 (21.6%)
OLBI scores	43 (38, 47)
Burnout	1239 (42.8%)
ISI scores	9 (5, 13)
Clinical insomnia	562 (19.4%)
QOL scores	6 (5, 8)
Low QOL	1069 (36.9%)
Turnover intention	651 (22.5%)
Suicidal ideation	853 (29.5%)

Abbreviations: GAD‐7, Generalized Anxiety Disorder Scale‐7; ISI, Insomnia Severity Index; OLBI, Oldenburg Burnout Inventory; PHQ‐9, Patient Health Questionnaire‐9; QOL, quality of life.

^a^Median (IQR); *n* (%)

### 3.2. The ABDI Network of HCWs

**Figure 1:**
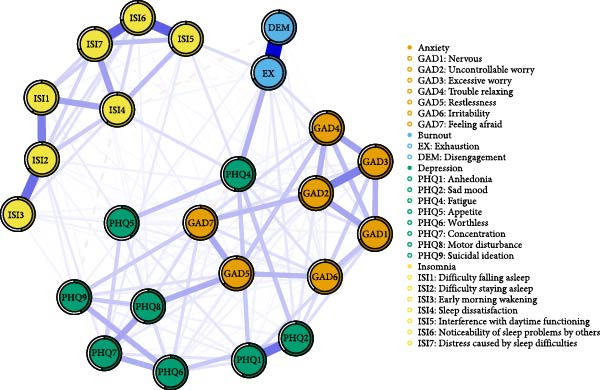
Network structure of anxiety, burnout, depression, and insomnia (ABDI) symptoms in healthcare workers. Edge thickness represents the degree of association; blue (solid) edges indicate positive relations, while red (dashed) edges indicate negative relationships. The colored area in the rings around the nodes depicts predictability (the variance of a given node explained by all its neighbors).

Table 2 presents the content of each item on the GAD‐7, OLBI, PHQ‐9, and ISI, along with their mean values (standard deviations), strength values, BS values, and predictability. Figure 1 illustrates the high‐density symptomatic network of ABDI, comprising 24 nodes and 176 nonzero edges, with an average edge weight of 0.041. The most substantial edges were observed between EX and DEM; interference with daytime functioning (ISI5) and noticeability of sleep problems by others (ISI6); and ISI6 and distress caused by sleep difficulties (ISI7). These edges showed significantly greater strength than others, as indicated by the bootstrap test results (Figure S1). Within the anxiety symptom community, the strongest edge was between uncontrollable worry (GAD2) and excessive worry (GAD3). In the depression symptom community, the most robust edge was between anhedonia (PHQ1) and sad mood (PHQ2). The strongest link between anxiety and depression symptoms was found between restlessness (GAD5) and motor disturbance (PHQ8). Similarly, the most prominent connection between burnout and depression symptoms was observed between EX and fatigue (PHQ4).

**Table 2:** Characteristics of each item on the GAD‐7, OLBI, PHQ‐9, and ISI.

Items	Content	Mean (SD)	Strength	BS	Predictability
GAD1	Nervous	1.12 (0.79)	0.30	0.34	0.70
GAD2	Uncontrollable worry	0.91 (0.88)	1.34	0.25	0.75
GAD3	Excessive worry	1.07 (0.86)	0.48	0.16	0.72
GAD4	Trouble relaxing	1.00 (0.87)	0.60	0.24	0.71
GAD5	Restlessness	0.63 (0.78)	0.53	0.50	0.64
GAD6	Irritability	1.03 (0.81)	0.03	0.42	0.61
GAD7	Feeling afraid	0.67 (0.80)	0.29	0.43	0.61
EX	Exhaustion	21.7 (4.12)	1.54	0.60	0.69
DEM	Disengagement	21.1 (3.94)	−1.51	0.18	0.60
PHQ1	Anhedonia	1.08 (0.77)	−0.51	0.20	0.57
PHQ2	Sad mood	1.03 (0.76)	0.13	0.33	0.62
PHQ4	Fatigue	1.51 (0.87)	0.35	0.59	0.54
PHQ5	Appetite	1.01 (0.89)	−1.74	0.28	0.41
PHQ6	Worthless	0.86 (0.85)	0.19	0.30	0.56
PHQ7	Concentration	0.95 (0.87)	−0.49	0.29	0.52
PHQ8	Motor disturbance	0.61 (0.80)	−0.34	0.42	0.54
PHQ9	Suicidal ideation	0.39 (0.69)	−1.50	0.39	0.43
ISI1	Difficulty falling asleep	1.12 (1.05)	−0.79	0.14	0.59
ISI2	Difficulty staying asleep	0.93 (1.01)	1.33	0.23	0.65
ISI3	Early morning wakening	1.11 (1.06)	−2.24	0.16	0.46
ISI4	Sleep dissatisfaction	2.00 (1.01)	0.42	0.18	0.61
ISI5	Interference with daytime functioning	1.59 (1.05)	−0.19	0.25	0.66
ISI6	Noticeability of sleep problems by others	1.39 (1.07)	1.22	0.08	0.73
ISI7	Distress caused by sleep difficulties	1.26 (1.11)	0.55	0.11	0.70

Abbreviations: BS, bridge strength; SD, standard deviation.

The burnout symptoms EX and DEM demonstrated varying degrees of association with depression, anxiety, and insomnia symptoms. EX was positively correlated with five out of eight depressive symptoms, four out of seven anxiety symptoms, and three out of seven insomnia symptoms, while it showed a negative correlation with three insomnia symptoms. In contrast, DEM exhibited positive associations with three depressive symptoms, two anxiety symptoms, and one insomnia symptom, while it was negatively correlated with one depressive symptom and one insomnia symptom. The estimated edge weights within the network were provided in Table S1.

### 3.3. Central and Bridge Symptoms

Figure 2A presents the centrality indices (strength) of each node in the ABDI network. It was observed that EX showed the highest centrality, followed by GAD2 and difficulty staying asleep (ISI2). Figure 2B depicts the BS of each node, with EX, PHQ4, and GAD5 identified as pivotal bridge symptoms driving the ABDI network. These symptoms exhibited notably higher strength or BS compared to other nodes (Figure S2A,B).

Figure 2The central and bridge symptoms of the ABDI network. (A) The centrality (strength) plot of the ABDI network. Nodes with higher strength have a stronger impact on other nodes within the network. (B) The bridge strength plot of the ABDI network. Nodes with higher bridge strength are recognized as bridge symptoms that drove the comorbidity. See Table 2 for full symptom names.

**Figure:**
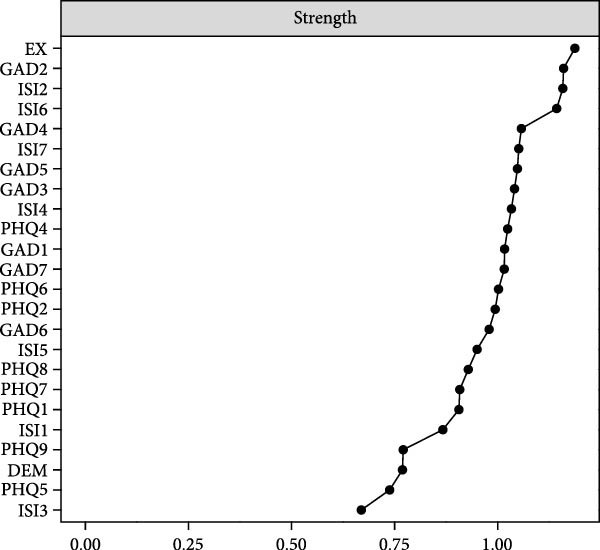
(A)

**Figure:**
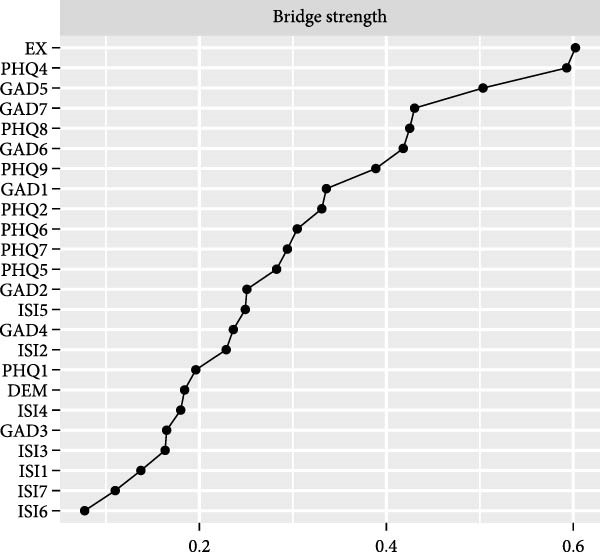
(B)

The average predictability of all nodes was 0.61, indicating that more than half of the variability in the symptoms could be predicted by other symptoms within the network. Notably, GAD2, ISI6, and GAD3 demonstrated the highest predictability, with values ranging from 0.72 to 0.75. In contrast, appetite (PHQ5) exhibited the lowest predictability, with a value of 0.41 (Table 2).

### 3.4. Stability and Accuracy of the Network

The results of bootstrapped differences between nodes (Figure S2A,B) or edge weights (Figure S1) suggested excellent stability and accuracy in the estimated network structure. The CS‐C values for strength and BS were both high, reaching 0.75 (Figure 3A), indicating that the network remained highly correlated with the original dataset even after dropping out 75% of the data. Additionally, the bootstrapped 95% CIs were narrow and nonoverlapping, suggesting the high accuracy of the estimated edges (Figure 3B).

Figure 3The stability and accuracy of the ABDI network. (A) The stability of central and bridge strength by case‐dropping bootstrap. The CS‐C for both the strength and bridge strength was 0.75. (B) The accuracy of the network edges by nonparametric bootstrapping. The gray area represents the bootstrap 95% confidence interval, which was narrow and indicated high accuracy.

**Figure:**
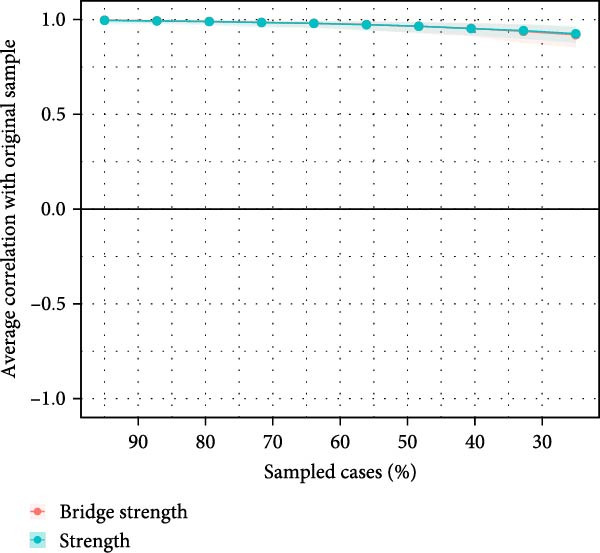
(A)

**Figure:**
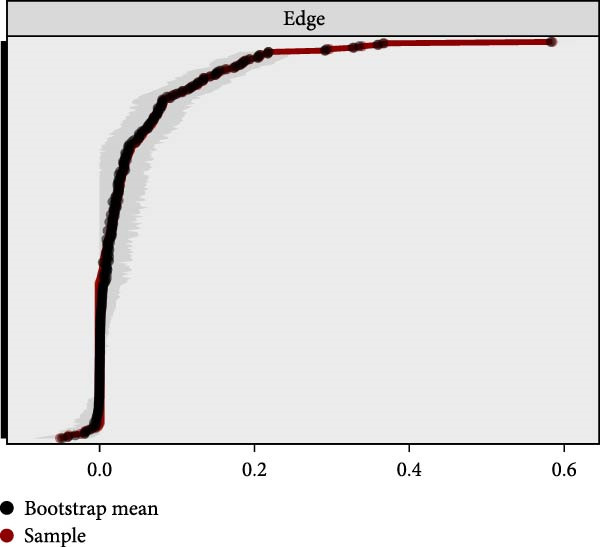
(B)

### 3.5. Symptoms Directly Related to Negative Consequences

Figure 4A–C illustrates the symptoms directly linked to adverse occupational and personal outcomes, including TI, low QOL, and SI. Specifically, TI was associated with 10 symptoms of mental distress, with DEM and PHQ9 (SI) showing notable positive correlations with TI. QOL was associated with 17 symptoms of mental distress, exhibiting strong negative associations with EX and sleep dissatisfaction (ISI4), as well as several depression and anxiety symptoms. SI was linked to 15 symptoms of mental distress, with the most robust connections observed for worthless (PHQ6), PHQ8, and feeling afraid (GAD7).

Figure 4Flow network of adverse occupational and personal outcomes for healthcare workers. It showed the symptoms directly related to these adverse outcomes. Blue edges indicate positive associations, while red edges indicate negative associations. Thicker edges imply stronger relationships. (A) Flow network of turnover intention. (B) Flow network of quality of life. (C) Flow network of suicidal ideation.

**Figure:**
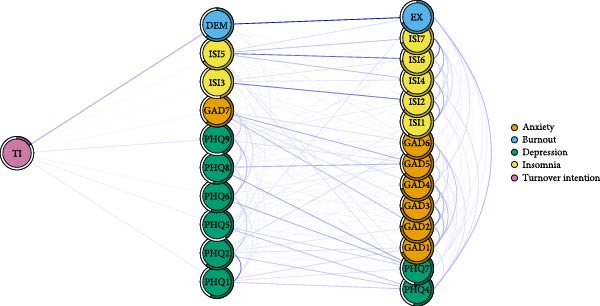
(A)

**Figure:**
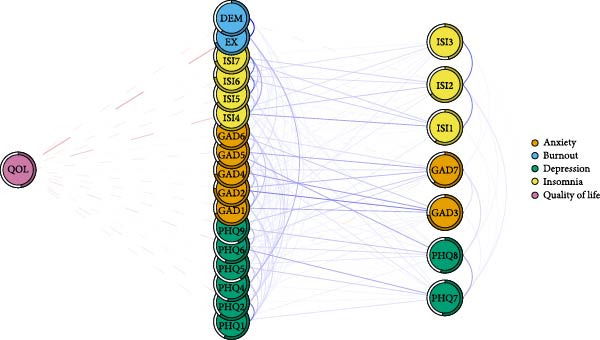
(B)

**Figure:**
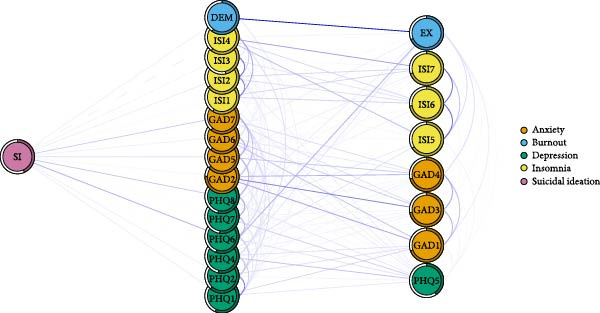
(C)

## 4. Discussion

This study is the first to use NA to examine the interactions among ABDI in HCWs exposed to WPV, as well as their associations with adverse outcomes—including SI, TI, and low QOL. The findings revealed distinct activation patterns in the ABDI network associated with WPV exposure. Our findings include the following: (1) The ABDI network was highly dense, indicating close relationships among depression, burnout, anxiety, and insomnia at the symptom level in WPV‐exposed HCWs. (2) EX served as the most central symptom and critical bridge symptom triggering and maintaining the ABDI network. (3) Adverse outcomes were strongly correlated with ABDI. DEM and PHQ9 were positively correlated with TI; EX, ISI4, and several depression and anxiety symptoms were negatively associated with QOL, while SI showed the closest correlations with PHQ6, PHQ8, and GAD7.

Our study highlighted the critical role of burnout in the network of mental distress and adverse outcomes among HCWs exposed to WPV. Previous studies have found that physicians experiencing verbal and physical violence report a statistically higher frequency of burnout symptoms [23, 56], and such violence is directly associated with higher burnout rates among nurses [57]. Loss of occupational performance has been found to be the most common outcome of exposure to violence, including changes in attitudes and decision‐making, as well as an increase in defensive medical behavior, which may further contribute to burnout [58, 59]. Burnout is characterized by negative emotional responses to chronic workplace stressors [60, 61], particularly in the context of exposure to WPV, reinforcing the association of burnout with other mental health problems such as anxiety, depression, and sleep disorders [62–64], and these effects are compounded [65]. Our team first identified EX as both the most central and bridge symptom in the ABDI network of HCWs exposed to WPV. Previous studies have shown that EX is the core dimension of burnout, with other dimensions serving as manifestations of EX or a continuation of this state [66, 67]. These findings align with our results, further underscoring EX’s pivotal role in the ABDI symptom network. In this study, DEM was found to be significantly positively correlated with TI, and EX showed a strong negative correlation with QOL. Consistent with previous research [68, 69], these results highlight that burnout is not limited to workplace‐related distress; it is also strongly associated with a range of adverse personal and occupational outcomes. Therefore, regular screening for burnout is recommended, particularly for HCWs who have experienced WPV, with a specific focus on their mental health and diminished QOL.

HCWs exposed to WPV face a significantly increased risk of developing psychological issues, such as depression and anxiety, leading to a reduced QOL, heightened TI, and, in severe cases, increased risk of suicide [23, 70, 71]. Our study found that anxiety and depressive symptoms not only interact with each other throughout the network but also exert a critical influence. Specifically, GAD2 was the central symptom, and PHQ4 and GAD5 were the bridge symptoms. Moreover, anxiety and depressive symptoms had significant effects on both adverse personal outcomes (SI, low QOL) and adverse occupational outcomes (TI) [72]. In our sample, the prevalence of depression and anxiety among WPV‐exposed HCWs was 36.1% and 21.6%, respectively, consistent with findings from a prior study of 3000 HCWs [73]. This aligns with other research showing that WPV is significantly associated with higher prevalence of anxiety and depression [20, 74]. Emerging evidence further suggests a bidirectional relationship between WPV and psychopathological symptoms. On the one hand, WPV triggers high levels of negative emotions and aggressive reactions, and promotes psychopathological symptoms [75]. On the other hand, uncontrolled and aberrant behavior in the workplace may exacerbate psychopathological symptoms and increase the risk of re‐exposure to WPV [35]. Such a vicious cycle will not only undermine clinical performance and increase turnover rates but also contribute to a further decline in the quality of healthcare and even lead to SI. Thus, it is essential to monitor the emotional responses of WPV‐exposed HCWs and provide targeted support to mitigate adverse mental health impacts. For example, after a WPV incident, interventions such as debriefing sessions, regular follow‐up check‐ins, and professional psychological support should be offered to validate affected individuals’ experiences and alleviate distress.

The relationship between WPV and sleep is of critical significance. Solid evidence already indicates that sleep problems are among the most common consequences of WPV experienced by HCWs [22, 70, 76]. In this study, we found that ISI2 was a central symptom in the ABDI network and that ISI4 was significantly negatively correlated with QOL. Consistent with previous studies, the present study found that nearly half of the HCWs exposed to WPV experienced insomnia [71]. A study conducted on Chinese doctors also found that exposure to WPV had a significant impact on both sleep quality and self‐reported health status [22]. WPV can be regarded as a stressful life event that may disrupt the sleep system due to sleep reactivity [77]. Sleep reactivity plays a key etiological role in the susceptibility to insomnia and other sleep disorders. The response to WPV, influenced by sleep reactivity, may manifest as dysregulation of parasympathetic activity, with symptoms including difficulty falling or maintaining sleep, irritability or angry outbursts, and difficulty concentrating [78, 79]. Sleep‐related problems could induce negative emotional states and may result in further adverse consequences. Therefore, we emphasize the need to assess both the quantity and quality of sleep in HCWs following exposure to WPV and to promote improved sleep hygiene.

Our study used a relatively large sample size and applied NA to identify symptom‐level associations. The following are several noteworthy implications. First, the study highlights the urgent need for continuous, systematic screening of WPV‐exposed HCWs for burnout, anxiety, depression, and insomnia. This proactive approach may help detect early signs of mental distress before symptoms escalate. Second, our study reveals strong associations between healthcare burnout, mental distress, and adverse personal and occupational outcomes under WPV. We further identified key symptoms (e.g., GAD2, ISI2, PHQ4, and GAD5) as potential drivers of the ABDI network. These symptoms serve as direct, actionable targets for intervention. For example, adequate sleep and appropriate physical activity may contribute to reducing fatigue and energy depletion [80]. Finally, EX is the most important symptom of the ABDI network and a strong predictor of low QOL. This suggests that burnout is not merely an occupational issue but has broader implications for the overall well‐being of WPV‐exposed HCWs. Timely identification and intervention for EX are therefore important; cognitive–behavioral therapy and mindfulness meditation may be helpful. These findings offer valuable guidance to healthcare organizations and hospital administrators aimed at minimizing the detrimental effects of exposure to occupational violence in HCWs.

Although the insights presented are valuable, this study has several limitations that should be acknowledged. First, as a cross‐sectional study, it cannot establish a causal relationship between WPV exposure and symptoms of burnout, depression, anxiety, or insomnia. Future longitudinal studies are therefore needed to validate the directional relationships observed in the present findings. Second, the online snowball sampling method restricted the representativeness of our sample. However, the incidence of mental distress and negative outcomes for HCWs exposed to WPV was pretty close to findings of other large‐scale studies of Chinese HCWs, suggesting that selection bias is unlikely [57, 73]. Third, regarding WPV surveys, it was not possible to assess the intensity of violence that occurred in the previous year or determine whether HCWs had received treatment. Fourth, it was challenging to objectively document episodes of violence due to underreporting. Furthermore, the requirement for respondents to answer all items may have introduced a selection bias. For future research in this sensitive area, incorporating a “prefer not to answer” option for sensitive items will enhance ethical rigor and reduce this potential bias. Finally, the use of a self‐reported questionnaire instead of a clinical interview to assess mental distress may have influenced the study outcomes. In conclusion, additional large‐scale, longitudinal, and representative studies are needed to confirm our findings.

## 5. Conclusions

In conclusion, this study establishes the first network of burnout, depression, anxiety, and insomnia among HCWs exposed to WPV. Our study highlights the importance of burnout in sustaining networks of mental distress, promoting comorbidities, and contributing to a reduced QOL. Burnout should be considered a priority for prevention and intervention efforts. We also provide evidence suggesting that burnout may be a phenomenon extending beyond the workplace. It is not only closely linked to mental distress but also strongly correlated with various adverse outcomes. Most of all, effective control strategies need to be further developed at the individual, hospital, and national levels to address WPV. When HCWs experience WPV, it is crucial to focus on their emotional responses and offer support to avoid adverse impacts.

## Disclosure

The funders had no further role in this study design, in the data collection and analysis, in the writing of the report, and in the decision to submit the paper for publication. All co‐authors revised and agreed to publish the final version of the manuscript.

## Conflicts of Interest

The authors declare no conflicts of interest.

## Author Contributions

Qiuxia Wu and Tieqiao Liu provided the design and supervision for this study. Xin Wang and Min Wu were responsible for data collection. Data analysis and interpretation were performed by Huixue Xu and Zejun Li. Huixue Xu and Zejun Li prepared the initial draft of the manuscript and critically revised by Qiuxia Wu and Tieqiao Liu. Huixue Xu and Zejun Li contributed equally to this work and should be listed as co‐first authors.

## Funding

This work was supported by National Natural Science Foundation of China (Grant No. 82371501 to Tieqiao Liu) and the Regional Innovation and Development Joint Fund of the National Natural Science Foundation (Grant No. U22A20302 to Tieqiao Liu).

## Supporting Information

Additional supporting information can be found online in the Supporting Information section.

## Supporting information


**Supporting Information** Table S1. illustrates the estimated edge weights within the ABDI network (Correlation matrix). Figure S1. illustrates the differences between edge weights in ABDI network derived from bootstrapped difference test. Figure S2. illustrates the nonparametric bootstrapped difference test of nodes in ABDI network. (A) Strength differences. (B) BS differences.

## Data Availability

The data that support the findings of this study are available from the corresponding author upon reasonable request.
