# Supplementary material for: Interrelationships of Anxiety, Burnout, Depression, and Insomnia Among Chinese Healthcare Workers Exposed to Workplace Violence: A Network Analysis
Source: Depress Anxiety. 2025 Dec 15;2025:9815503. doi: 10.1155/da/9815503 (PMC12714119; doi:10.1155/da/9815503)
Supplement: Supplementary file 1 — Supporting Information Table S1. illustrates the estimated edge weights within the ABDI network (Correlation matrix). Figure S1. illustrates the differences between edge weights in ABDI network derived from bootstrapped difference test. Figure S2. illustrates the nonparametric bootstrapped difference test of nodes in ABDI network. (A) Strength differences. (B) BS differences. [file DA-2025-9815503-s001.doc]

| **Table S1. The estimated edge weights within the ABDI network (Correlation matrix)** | | | | | | | | | | | | | | | | | | | | | | | | |
| --- | --- | --- | --- | --- | --- | --- | --- | --- | --- | --- | --- | --- | --- | --- | --- | --- | --- | --- | --- | --- | --- | --- | --- | --- |
|  | PHQ1 | PHQ2 | PHQ4 | PHQ5 | PHQ6 | PHQ7 | PHQ8 | PHQ9 | GAD1 | GAD2 | GAD3 | GAD4 | GAD5 | GAD6 | GAD7 | ISI1 | ISI2 | ISI3 | ISI4 | ISI5 | ISI6 | ISI7 | EX | DEM |
| PHQ1 | 0 | 0.329 | 0.127 | 0.056 | 0.062 | 0.067 | 0.032 | 0.036 | 0.090 | 0.021 | 0.002 | 0.008 | 0.009 | 0.022 | 0 | 0 | 0 | 0 | 0 | 0.003 | 0 | 0 | 0.0115 | 0.030 |
| PHQ2 | 0.329 | 0 | 0.133 | 0.024 | 0.126 | 0.019 | 0 | 0.032 | 0.075 | 0.059 | 0.0278 | 0.024 | 0 | 0.082 | 0 | 0 | 0 | 0.003 | 0 | 0 | 0.006 | 0 | 0.054 | 0 |
| PHQ4 | 0.127 | 0.133 | 0 | 0.143 | 0 | 0.022 | 0 | -0.006 | 0.073 | 0 | 0.032 | 0.034 | -0.019 | 0.070 | -0.051 | 0 | 0 | 0 | 0.075 | 0.062 | 0 | 0 | 0.176 | 0 |
| PHQ5 | 0.056 | 0.024 | 0.143 | 0 | 0.069 | 0.107 | 0.056 | 0 | 0.027 | 0.014 | 0.025 | 0.015 | 0.014 | 0.039 | 0.026 | 0.041 | 0.005 | 0.037 | 0 | 0.022 | 0 | 0.006 | 0.010 | 0 |
| PHQ6 | 0.0627 | 0.126 | 0 | 0.069 | 0 | 0.186 | 0.080 | 0.174 | 0.010 | 0.032 | 0.027 | 0.025 | 0.046 | 0.016 | 0.083 | 0 | 0 | 0.007 | 0 | 0 | 0 | 0.021 | 0 | 0.036 |
| PHQ7 | 0.067 | 0.019 | 0.022 | 0.107 | 0.186 | 0 | 0.207 | 0.006 | 0.012 | 0.007 | 0.027 | 0.026 | 0.055 | 0.002 | 0.009 | 0.017 | 0.050 | 0.006 | 0 | 0.048 | 0 | 0 | 0.038 | 0 |
| PHQ8 | 0.032 | 0 | 0 | 0.056 | 0.080 | 0.207 | 0 | 0.129 | 0 | 0.026 | 0 | 0.004 | 0.188 | 0.025 | 0.081 | 0.014 | 0.026 | 0 | -0.014 | 0 | 0.030 | 0.017 | 0 | -0.000 |
| PHQ9 | 0.036 | 0.032 | -0.006 | 0 | 0.174 | 0.006 | 0.129 | 0 | 0 | 0.020 | 0 | 0 | 0.070 | 0.032 | 0.118 | 0.011 | 0.040 | 0.025 | -0.042 | 0 | 0 | 0 | 0 | 0.031 |
| GAD1 | 0.090 | 0.075 | 0.073 | 0.027 | 0.010 | 0.012 | 0 | 0 | 0 | 0.205 | 0.182 | 0.153 | 0 | 0.136 | 0.005 | 0 | 0 | 0 | 0 | 0 | 0 | 0 | 0.043 | 0.006 |
| GAD2 | 0.021 | 0.059 | 0 | 0.014 | 0.032 | 0.007 | 0.026 | 0.020 | 0.205 | 0 | 0.292 | 0.183 | 0.051 | 0.020 | 0.157 | 0 | 0.032 | 0.021 | 0 | 0 | 0 | 0.018 | 0 | 0 |
| GAD3 | 0.002 | 0.028 | 0.032 | 0.025 | 0.027 | 0.027 | 0 | 0 | 0.182 | 0.292 | 0 | 0.217 | 0 | 0.1144 | 0.070 | 0 | 0 | 0 | 0 | 0.011 | 0 | 0 | 0.013 | 0 |
| GAD4 | 0.008 | 0.024 | 0.034 | 0.015 | 0.025 | 0.026 | 0.004 | 0 | 0.153 | 0.183 | 0.217 | 0 | 0.135 | 0.050 | 0.083 | 0 | 0 | 0 | 0 | 0.006 | 0.015 | 0.004 | 0.076 | 0 |
| GAD5 | 0.009 | 0 | -0.019 | 0.014 | 0.046 | 0.055 | 0.188 | 0.070 | 0 | 0.051 | 0 | 0.135 | 0 | 0.164 | 0.194 | 0.017 | 0.019 | 0.015 | 0 | 0 | 0.016 | 0.035 | 0 | 0 |
| GAD6 | 0.022 | 0.082 | 0.070 | 0.039 | 0.016 | 0.002 | 0.025 | 0.032 | 0.136 | 0.020 | 0.114 | 0.050 | 0.164 | 0 | 0.077 | 0.012 | 0 | 0.028 | 0 | 0.017 | 0 | 0 | 0.039 | 0.033 |
| GAD7 | 0 | 0 | -0.051 | 0.026 | 0.083 | 0.009 | 0.081 | 0.118 | 0.005 | 0.157 | 0.070 | 0.083 | 0.194 | 0.076 | 0 | 0 | 0.016 | 0.019 | -0.019 | 0 | 0 | 0.008 | 0 | 0 |
| ISI1 | 0 | 0 | 0 | 0.041 | 0 | 0.017 | 0.014 | 0.011 | 0 | 0 | 0 | 0 | 0.017 | 0.012 | 0 | 0 | 0.297 | 0.003 | 0.208 | 0.019 | 0.108 | 0.096 | -0.025 | 0 |
| ISI2 | 0 | 0 | 0 | 0.005 | 0 | 0.050 | 0.026 | 0.040 | 0 | 0.032 | 0 | 0 | 0.019 | 0 | 0.016 | 0.297 | 0 | 0.339 | 0.119 | 0 | 0.075 | 0.100 | -0.002 | -0.040 |
| ISI3 | 0 | 0.003 | 0 | 0.037 | 0.007 | 0.006 | 0 | 0.025 | 0 | 0.021 | 0 | 0 | 0.015 | 0.028 | 0.019 | 0.003 | 0.340 | 0 | 0.067 | 0.013 | 0.065 | 0.018 | -0.002 | 0 |
| ISI4 | 0 | 0 | 0.075 | 0 | 0 | 0 | -0.014 | -0.042 | 0 | 0 | 0 | 0 | 0 | 0 | -0.019 | 0.208 | 0.119 | 0.067 | 0 | 0.152 | 0.090 | 0.218 | 0.023 | 0.007 |
| ISI5 | 0.003 | 0 | 0.062 | 0.022 | 0 | 0.048 | 0 | 0 | 0 | 0 | 0.011 | 0.006 | 0 | 0.017 | 0 | 0.019 | 0 | 0.013 | 0.152 | 0 | 0.368 | 0.150 | 0.080 | 0 |
| ISI6 | 0 | 0.006 | 0 | 0 | 0 | 0 | 0.030 | 0 | 0 | 0 | 0 | 0.015 | 0.016 | 0 | 0 | 0.108 | 0.075 | 0.065 | 0.090 | 0.368 | 0 | 0.360 | 0.010 | 0 |
| ISI7 | 0 | 0 | 0 | 0.006 | 0.021 | 0 | 0.017 | 0 | 0 | 0.018 | 0 | 0.004 | 0.035 | 0 | 0.008 | 0.096 | 0.100 | 0.018 | 0.218 | 0.150 | 0.360 | 0 | 0 | 0 |
| EX | 0.011 | 0.054 | 0.176 | 0.010 | 0 | 0.038 | 0 | 0 | 0.043 | 0 | 0.013 | 0.076 | 0 | 0.039 | 0 | -0.025 | -0.002 | -0.002 | 0.023 | 0.080 | 0.010 | 0 | 0 | 0.585 |
| DEM | 0.030 | 0 | 0 | 0 | 0.036 | 0 | -0.000 | 0.031 | 0.006 | 0 | 0 | 0 | 0 | 0.033 | 0 | 0 | -0.040 | 0 | 0.007 | 0 | 0 | 0 | 0.585 | 0 |

ABDI: anxiety, burnout, depression and insomnia; EX: exhaustion; DEM: disengagement.

Figure S1. Differences between edge weights in anxiety, burnout, depression, and insomnia (ABDI) network derived from bootstrapped difference test.

1. **(B)**

Figure S2. Nonparametric bootstrapped difference test of nodes in ABDI network. (A) Strength differences. (B) Bridge strength differences.
